# Latent profiles of kinesiophobia and associated factors in patients with rheumatoid arthritis: a cross-sectional study

**DOI:** 10.3389/fmed.2026.1868172

**Published:** 2026-07-31

**Authors:** Yanli You, Nor Aziyan Yahaya, Vimala Ramoo, Fariz Yahya, Xiaofei Shi, Xueying Shang, Wei Xu, Dandan Jiao

**Affiliations:** 1 Department of Nursing Science, Faculty of Medicine, University Malaya, Kuala Lumpur, Malaysia; 2 Rheumatology Unit, The First Affiliated Hospital, and College of Clinical Medicine of Henan University of Science and Technology, Luoyang, Henan, China; 3 School of Nursing, Faculty of Health Sciences, Nursing and Education, Mahsa University, Selangor, Malaysia; 4 Rheumatology, Faculty of Medicine, University Malaya, Kuala Lumpur, Malaysia

**Keywords:** fear of movement, kinesiophobia, latent profile analysis, profile characteristics, rheumatoid arthritis

## Abstract

**Background:**

Patients with rheumatoid arthritis (RA) frequently experience kinesiophobia, driven by concerns that movement may worsen pain or cause joint damage. This fear can reduce physical activity and adversely affect functional outcomes. Conventional approaches that classify patients solely based on overall kinesiophobia scores may fail to capture important differences in symptom presentation and behavioral responses.

**Objective:**

This study aimed to identify distinct latent profiles of kinesiophobia among patients with RA using the Tampa Scale of Kinesiophobia-11 (TSK-11) and to compare subgroup characteristics to inform targeted, individualized intervention strategies.

**Methods:**

A convenience sample of patients with RA was recruited from three tertiary hospitals in Henan Province between May 1 and June 30, 2023. Data collected included demographic characteristics and responses to the Tampa Scale of Kinesiophobia-11 (TSK-11), Brief Pain Inventory-Short Form (BPI-SF), Pain Anxiety Symptoms Scale (PASS), Patient Activation Measure (PAM), Social Support Rating Scale (SSRS), International Physical Activity Questionnaire-Short Form (IPAQ-SF), and WHOQOL-BREF. Latent profile analysis was performed using Mplus 8.0 to identify distinct kinesiophobia profiles, followed by univariate analyses and *post hoc* comparisons in SPSS 27.0.

**Results:**

A total of 604 patients with RA were enrolled, with a mean age of 57.04 ± 12.52 years; 76.8% were female. Four distinct kinesiophobia profiles were identified: Class 1, low cognition-attitude with moderate behavior (13.7%); Class 2, low cognition with high attitude-behavior (18.0%); Class 3, high cognition with high attitude-behavior (60.8%); and Class 4, extremely high cognition-attitude-behavior (7.5%). Significant differences were observed among the profiles in gender, monthly income, disease activity, physical activity, pain intensity, pain interference, quality of life, social support, pain anxiety, and patient activation. *Post hoc* analyses indicated that Class 4 showed the greatest pain intensity, pain interference, and pain anxiety, along with the highest proportion of low physical activity and the lowest level of social support. Class 1 demonstrated more favorable disease status, quality of life, and social support, while patient activation was significantly higher in Classes 2 and 3 than in Class 1.

**Conclusion:**

Kinesiophobia among patients with RA is heterogeneous, with distinct profiles differing in sociodemographic, clinical, psychosocial, and self-management characteristics. Latent profile analysis provides a useful approach for identifying cognitive, attitudinal, and behavioral subgroups, facilitating the development of personalized interventions to reduce fear of movement and encourage safe participation in physical activity.

## 1 Introduction

Rheumatoid arthritis (RA) is a chronic autoimmune disease characterized by persistent inflammation, pain, and progressive functional impairment, all of which substantially reduce patients' quality of life (1). Regular physical activity is a fundamental component of RA management because of its well-established benefits for joint function, cardiovascular health, and overall wellbeing (2). Despite these recognized benefits, many patients remain insufficiently active and spend prolonged periods in sedentary behavior (3), underscoring a persistent gap between evidence-based recommendations and actual clinical practice.

Psychological barriers have emerged as important contributors to this discrepancy. Among them, kinesiophobia, defined as an excessive and debilitating fear of movement resulting from concerns about pain or injury, is highly prevalent in patients with RA and has consistently been linked to reduced physical activity and poorer functional outcomes (4). The Fear-Avoidance Model proposes that maladaptive interpretations of pain can trigger fear and avoidance behaviors, eventually leading to physical deconditioning and disability (5). However, this process varies considerably among individuals, with patients differing in how they perceive, interpret, and respond to pain-related experiences.

Most previous studies have assessed kinesiophobia using total questionnaire scores and variable-centered analytical approaches, which assume that the study population is relatively homogeneous (4, 6). Such methods may obscure meaningful interindividual differences and hinder the identification of clinically important subgroups with distinct psychological and behavioral characteristics (7). Therefore, interventions developed from average population effects may not adequately address the diverse needs of different patient subgroups.

Person-centered methods, such as latent profile analysis (LPA), offer an alternative approach by identifying unobserved subgroups according to individuals' response patterns rather than overall scale scores (8). By capturing the heterogeneity of kinesiophobia, LPA can provide a more nuanced understanding of patient subtypes and their associated characteristics, facilitating the design of more personalized intervention strategies. However, the application of LPA to kinesiophobia in patients with RA remains limited, particularly regarding the combined influence of psychosocial and clinical factors on profile membership.

Therefore, this study aimed to (1) identify latent profiles of kinesiophobia among patients with RA using LPA and (2) examine the demographic, clinical, and psychosocial factors associated with these profiles, with the ultimate goal of informing more targeted and individualized strategies for the management of kinesiophobia.

## 2 Materials and methods

### 2.1 Ethical considerations

This study was approved by the Ethics Committee of the First Affiliated Hospital of Henan University of Science and Technology (Approval No. 2023–687) and was conducted in accordance with the principles of the Declaration of Helsinki. Written informed consent was obtained from all participants before enrollment.

### 2.2 Participants and recruitment

Convenience sampling was used to recruit patients with RA. Participants were consecutively enrolled from the rheumatology outpatient clinics of three tertiary Grade A hospitals in Henan Province, China, between May 1 and June 30, 2023. The inclusion criteria were as follows: (1) fulfillment of the 2010 American College of Rheumatology/European League Against Rheumatism (ACR/EULAR) classification criteria for RA (9) with a confirmed diagnosis by a rheumatology specialist; (2) disease duration of at least 3 months; (3) age ≥18 years; (4) ability to complete the questionnaires independently or with minimal assistance; and (5) voluntary participation with written informed consent. The exclusion criteria were: (1) coexistence of other rheumatic or autoimmune diseases that could affect mobility or interfere with symptom interpretation, such as systemic lupus erythematosus, ankylosing spondylitis, Sjögren's syndrome, or psoriatic arthritis; (2) severe systemic diseases that substantially restricted mobility, including post-stroke sequelae, advanced heart failure, severe chronic obstructive pulmonary disease (COPD), or major neuromuscular disorders; (3) major surgery within the previous 3 months; and (4) cognitive impairment or psychiatric disorders that prevented valid completion of the questionnaires.

### 2.3 Sample size determination

The sample size was determined based on methodological recommendations for LPA. Because LPA requires a sufficiently large sample to ensure stable parameter estimation, accurate class identification, and adequate representation of latent subgroups, a sample size of 500 participants or more was considered appropriate for this study (7). A total of 723 questionnaires were distributed, and 604 valid responses were retained for the final analysis, resulting in an effective response rate of 83.5%.

### 2.4 Measures

The following instruments and questionnaires were used in this study:

#### 2.4.1 General information questionnaire

Demographic and clinical characteristics were collected, including age, sex, body mass index (BMI), marital status, monthly income, comorbidities, and medication use. Disease activity was evaluated using the Disease Activity Score in 28 joints (DAS28). Disease remission was defined as a DAS28 score < 2.6; low disease activity as 2.6 to < 3.2; moderate disease activity as 3.2–5.1; and high disease activity as >5.1 (6).

#### 2.4.2 Tampa scale of kinesiophobia−11 (TSK-11)

Kinesiophobia was assessed using the TSK-11 (10). The scale comprises 11 items rated on a 4-point Likert scale (1 = strongly disagree to 4 = strongly agree), with total scores ranging from 11 to 44. Higher scores indicate greater levels of kinesiophobia (11). The scale includes three dimensions: activity cognition (items 2–6 and 8), activity behavior (items seven, nine, and 10), and activity attitude (items one and 11) (12). The Chinese version has demonstrated good reliability and validity, with a reported Cronbach's α of 0.883 (13). In this study, the Cronbach's α coefficient was 0.860, indicating good internal consistency.

#### 2.4.3 Brief Pain Inventory–short form (BPI-SF)

Pain intensity and pain interference were measured using the BPI-SF, which employs an 11-point numeric rating scale (0–10) (14). Pain intensity was calculated as the average of the worst, least, average, and current pain experienced during the previous 24 h. Pain interference was assessed across several domains, including daily activities, work, sleep, mood, and social functioning (15). In this study, the Cronbach's α coefficients were 0.860 for pain intensity and 0.905 for pain interference.

#### 2.4.4 Pain anxiety symptoms scale (PASS)

Pain-related fear and anxiety were evaluated using the PASS (16). The scale consists of 20 items rated on a 6-point Likert scale from 0 (never) to 5 (always), yielding a total score ranging from 0 to 100, with higher scores reflecting greater pain-related anxiety (17). The Chinese version has demonstrated good reliability, with a reported Cronbach's α of 0.920 (18). In this study, the Cronbach's α coefficient was 0.953, indicating excellent internal consistency.

#### 2.4.5 Patient activation measure (PAM-13)

Patient activation was assessed using the PAM-13, which evaluates participants' knowledge, skills, and confidence in managing their health and disease (19). The instrument includes 13 items rated on a 4-point Likert scale from “strongly disagree” to “strongly agree,” with an additional “not applicable” response option (20). Raw scores were multiplied by 13 and converted to standardized scores ranging from 0 to 100 according to the revised scoring conversion table provided by Insignia Health. Higher scores indicate greater patient activation (21). The Cronbach's α coefficient in this study was 0.885.

#### 2.4.6 Social support rating scale (SSRS)

Social support was measured using the SSRS developed by Xiao Shuiyuan, which has been widely used in Chinese populations and has demonstrated satisfactory psychometric properties (22). The scale contains 10 items across three dimensions: subjective support (four items), objective support (three items), and support utilization (three items) (23). Total scores range from 12 to 66, with higher scores indicating stronger perceived social support (24). In this study, the Cronbach's α coefficient was 0.785, indicating acceptable internal consistency.

#### 2.4.7 International physical activity questionnaire–short form (IPAQ-SF)

Physical activity was assessed using the IPAQ-SF (25). Total physical activity was calculated by multiplying the metabolic equivalent of task (MET) values assigned to activities of different intensities by the duration (minutes/day) and frequency (days/week) of participation. It was expressed as MET-minutes per week (26). Based on international scoring guidelines, physical activity levels were classified as low, moderate, or high (25). The Chinese version of the IPAQ-SF has demonstrated good reliability, with a test–retest intraclass correlation coefficient (ICC) of ≥0.75 (27).

#### 2.4.8 World health organization quality of life–brief version (WHOQOL-BREF)

Quality of life was assessed using the WHOQOL-BREF, developed by the World Health Organization through international expert collaboration (7). The instrument consists of 26 items covering four domains: physical health, psychological health, social relationships, and environment (28). Domain scores were calculated according to the standardized WHO scoring procedures and transformed to a 0–100 scale, with higher scores indicating better quality of life (7). In this study, the Cronbach's α coefficients for the physical health, psychological health, social relationships, and environment domains were 0.803, 0.964, 0.891, and 0.877, respectively, indicating good internal consistency.

### 2.5 Procedure

Before data collection, institutional approval was obtained, and the head nurses of the participating wards were informed about the study procedures. Trained researchers screened eligible participants, explained the study objectives, and obtained written informed consent before enrollment. Participants completed the questionnaires in a quiet environment within 24 h. To ensure data quality, several quality control measures were implemented. Questionnaires containing more than 20% missing data, incomplete responses to key measures, or obvious response patterns indicating insufficient engagement were excluded from the final analysis.

### 2.6 Statistical analysis

Data were analyzed using Mplus 8.0 and SPSS 27.0. Missing data (< 5%) were handled using full-information maximum likelihood (FIML) for the latent profile analysis (LPA), whereas complete-case analysis was used for all other analyses. Harman's single-factor test was performed to evaluate the presence of common method bias.

Latent profile models with 1 to 5 classes were estimated sequentially. Model selection was based on multiple fit indices, including the Akaike information criterion (AIC), Bayesian information criterion (BIC), adjusted Bayesian information criterion (aBIC), entropy, and the significance of the Lo-Mendell-Rubin (LMR) test and bootstrap likelihood ratio test (BLRT). The optimal model was selected by considering statistical fit, classification accuracy, interpretability, and clinical relevance.

Descriptive data are presented as mean ± standard deviation (SD) or frequency and percentage (%), as appropriate. Differences between groups were assessed using one-way analysis of variance (ANOVA), chi-square tests, or Fisher's exact tests, where appropriate. When overall group differences were significant, Tukey's honestly significant difference (HSD) *post hoc* tests were conducted for continuous variables following one-way ANOVA. For categorical variables, Bonferroni-adjusted pairwise comparisons were conducted following χ^2^ or Fisher's exact tests, as appropriate. Effect sizes were quantified using eta-squared (η^2^) for continuous variables and Cramer's V for categorical variables. All statistical tests were two-sided, and *p*-values < 0.05 were considered statistically significant.

## 3 Results

### 3.1 Demographic and clinical characteristics

A total of 604 patients with RA were included in the final analysis. The mean age of the participants was 57.04 ± 12.52 years, and 76.8% (*n* = 464) were female. More than half (53.5%) had a normal body mass index, whereas 35.7% were classified as overweight or obese. The majority were married or living with a partner (92.9%), and 59.8% reported low levels of physical activity. The detailed demographic and clinical characteristics of the study population are summarized in Table 1.

**Table 1:** Demographic and clinical characteristics of RA participants (total sample *N* = 604).

Variables	Category	Mean ±SD/*n*	%
Age (years)		57.04 ± 12.52	
Gender	Male	140	23.2
	Female	464	76.8
BMI	Low	65	10.8
	Normal	323	53.5
	Overweight/obese	216	35.7
Marital status	Married/partnered	561	92.9
	Others (Lone/divorce/widow)	43	7.1
Education level	Elementary school and below	153	25.3
	Middle school	231	38.2
	High school	122	20.2
	Junior college and above	98	16.2
Occupation	Employed	102	16.9
	Retired	184	30.5
	Others	318	52.6
Monthly income (Chinese yuan)	< 2,000	289	47.8
	2,000–4,000	244	40.4
	>4,000	71	11.8
Comorbidity number		0.44 ± 0.72	
Medication	Biologicals	83	13.7
	Non-biologicals	521	86.3
History of fall	None	567	93.9
	Have	37	6.1
DAS28	Disease remission	132	21.9
	Low disease activity	117	19.4
	Moderate disease activity	261	43.2
	High disease activity	94	15.6
Physical activity (IPAQ-SF)	Low	361	59.8
	Moderate	229	37.9
	High	14	2.3
Pain intensity (BPI-SF)		3.38 ± 2.87	
Pain interference (BPI-SF)		15.28 ± 15.65	
Pain duration (days)		62.78 ± 76.35	
Physical health (WHOQOL-BREF)		54.66 ± 16.71	
Psychological health (WHOQOL-BREF)		54.84 ± 18.23	
Social relationships (WHOQOL-BREF)		54.09 ± 19.36	
Environment (WHOQOL-BREF)		54.90 ± 15.20	
Social support (SSRS)		35.42 ± 5.54	
Pain anxiety (PASS)		40.68 ± 22.16	
Kinesiophobia (TSK-11)		28.12 ± 6.28	
Patient activation (PAM-13)		63.47 ± 14.84	

BPI-SF, Brief Pain Inventory–short form; WHOQOL-BREF, world health organization quality of life-brief version; SSRS, Social Support Rating Scale; PASS, Pain Anxiety Symptoms Scale; TSK-11, Tampa Scale of Kinesiophobia-11; PAM-13, patient activation measure; IPAQ-SF, International Physical Activity Questionnaire-short form; BMI, Body Mass Index; DAS28, disease activity score in 28 joints; SD, standard deviation.

### 3.2 Latent profile of kinesiophobia in patients with RA

The fit indices for the one- to five-class latent profile models are presented in Table 2. The AIC, BIC, and aBIC decreased progressively as the number of classes increased, indicating improved model fit. Entropy values remained consistently high (≥0.950), demonstrating excellent classification accuracy. In addition, both the Lo-Mendell-Rubin (LMR) test and the bootstrap likelihood ratio test (BLRT) were statistically significant (*p* < 0.05) for all models.

**Table 2:** Latent profile analysis model fit indices for kinesiophobia in RA patients (*n* = 604).

Model	Log (L)	AIC	BIC	aBIC	Entropy	*p*-value		Latent class prevalence
						BLRT	LMR	
1	−8,367.593	16,779.186	16,876.065	16,806.220	—	—	—	1.000
2	−7,304.421	14,676.842	14,826.564	14,718.622	0.950	< 0.001	< 0.001	0.303/0.697
3	−6,983.475	14,058.951	14,261.515	14,115.477	0.968	< 0.001	< 0.001	0.137/0.186/0.677
4	−6,700.394	13,516.787	13,772.194	13,588.059	0.967	< 0.001	< 0.001	0.137/0.180/0.608/0.075
5	−6,558.463	13,256.925	13,565.176	13,342.943	0.966	0.002	< 0.001	0.054/0.083/0.179/0.611/0.073

AIC, akaike information criterion; BIC, Bayesian information criterion; aBIC, sample-adjusted BIC; BLRT, bootstrap likelihood ratio test; LMR, Lo-Mendell-Rubin test.

Although the five-class model showed further reductions in the fit indices, its smallest subgroup comprised only 33 patients (5.4% of the total sample). Such a small class may compromise parameter stability and limit the clinical applicability of the identified profiles. Therefore, considering statistical fit, model parsimony, classification performance, and clinical interpretability, the four-class model was selected as the optimal solution (AIC = 13,516.787, BIC = 13,772.194, aBIC = 13,588.059, entropy = 0.967).

**Figure 1:**
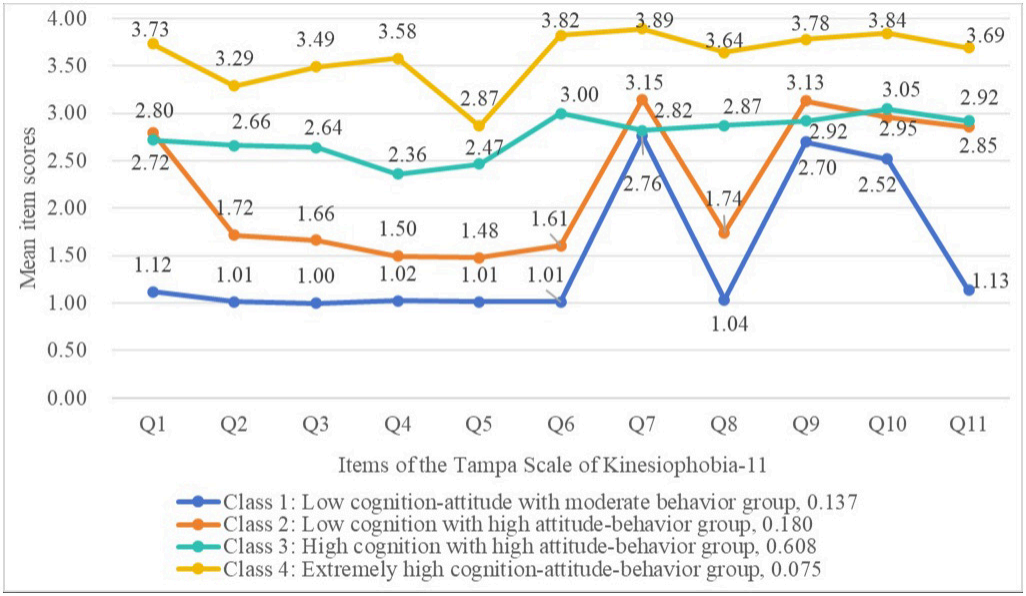
Item-level profiles of kinesiophobia across four latent classes.

Based on participants' response patterns on the TSK-11, four distinct latent profiles of kinesiophobia were identified in Figure 1. The item mean scores for each profile are presented in Supplementary Table S1, and the mean scores for each dimension are summarized in Supplementary Table S2.

*Class 1* (Low cognition-attitude with moderate behavior group; *n* = 83, 13.7%) comprised patients with low scores on the cognitive and attitudinal dimensions but moderate scores on the behavioral dimension.

*Class 2* (Low cognition with high attitude-behavior group; *n* = 109, 18.0%) included patients who demonstrated low cognitive scores but high attitudinal and behavioral scores.

*Class 3* (High cognition with high attitude-behavior group; *n* = 367, 60.8%) was the largest subgroup and was characterized by significantly elevated cognitive, attitudinal, and behavioral scores.

*Class 4* (Extremely high cognition-attitude-behavior group; *n* = 45, 7.5%) included patients with the highest scores across all three dimensions, cognition, attitude, and behavior, indicating the greatest overall level of kinesiophobia among all identified profiles.

### 3.3 Univariate analysis of latent kinesiophobia classes

Univariate analyses revealed significant differences among the four latent profiles in gender, monthly income, disease activity, physical activity, pain intensity, pain interference, quality of life, social support, pain anxiety, and patient activation (all *p* < 0.05), as presented in Table 3. Class 4 was characterized by the highest proportions of female participants, individuals with low monthly income, high disease activity, and low physical activity. This group also demonstrated the greatest pain intensity, pain interference, and pain anxiety, together with the lowest mean level of social support. *Post hoc* analyses demonstrated that both pain intensity and pain interference were significantly higher in Class 4 than in the other three profiles. Pain anxiety increased progressively from Class 1 to Class 4, with all pairwise comparisons reaching statistical significance.

**Table 3:** Univariate analysis of latent kinesiophobia profiles in RA patients (*N* = 604).

Variables category	Class 1 (*n* = 83)	Class 2 (*n* = 109)	Class 3 (*n* = 367)	Class 4 (*n* = 45)	F/χ^2^	*p*	Effect size
Age (years)	57.76 ± 13.30	56.40 ± 12.82	57.04 ± 12.25	57.33 ± 12.80	0.193	0.901	0.001
Gender							
Male	26 (31.3)^a^	26 (23.9)^a, b^	84 (22.9)^a, b^	4 (8.9)^b^	8.299	0.040	0.117
Female	57 (68.7)^a^	83 (76.1)^a, b^	283 (77.1)^a, b^	41 (91.1)^b^			
BMI							
Low	11 (13.3)	8 (7.3)	41 (11.1)	5 (11.1)	5.060	0.536	0.065
Normal	43 (51.8)	66 (60.6)	187 (51.0)	27 (60.0)			
Overweight/obese	29 (34.9)	35 (32.1)	139 (37.9)	13 (28.9)			
Marital status							
Married/partnered	78 (94.0)	100 (91.7)	344 (93.7)	39 (86.7)	3.395	0.335	0.075
Others (Lone/divorce/widow)	5 (6.0)	9 (8.3)	23 (6.3)	6 (13.3)			
Education level							
Elementary school and below	19 (22.9)	26 (23.9)	92 (25.1)	16 (35.6)	10.869	0.285	0.077
Middle school	31 (37.3)	42 (38.5)	143 (39.0)	15 (33.3)			
High school	16 (19.3)	16 (14.7)	82 (22.3)	8 (17.8)			
Junior college and above	17 (20.5)	25 (22.9)	50 (13.6)	6 (13.3)			
Occupation							
Employed	14 (16.9)	21 (19.3)	62 (16.9)	5 (11.1)	5.186	0.520	0.066
Retired	32 (38.5)	29 (26.6)	110 (30.0)	13 (28.9)			
Others	37 (44.6)	59 (54.1)	195 (53.1)	27 (60.0)			
Monthly income (Chinese yuan)							
< 2,000	24 (28.9)^a^	53 (48.6)^b^	182 (49.6)^b^	30 (66.7)^b^	28.171	< 0.001	0.153
2,000–4,000	40 (48.2)^a^	39 (35.8)^a^	151 (41.1)^a^	14 (31.1)^a^			
>4,000	19 (22.9)^a^	17 (15.6)^a, b^	34 (9.3)^b^	1 (2.2)^b^			
Comorbidity number	0.31 ± 0.56	0.42 ± 0.71	0.46 ± 0.72	0.51 ± 0.92	1.137	0.333	0.006
Medication							
Biologicals	17 (20.5)	17 (15.6)	40 (10.9)	9 (20.0)	7.486	0.058	0.111
Non-biologicals	66 (79.5)	92 (84.4)	327 (89.1)	36 (80.0)			
History of fall							
None	77 (92.8)	105 (96.3)	345 (94.0)	40 (88.9)	3.275	0.351	0.074
Have	6 (7.2)	4 (3.7)	22 (6.0)	5 (11.1)			
DAS28							
Disease remission	33 (39.8)^a^	26 (23.9)^a, b^	70 (19.1)^b^	3 (6.7)^b^	46.025	< 0.001	0.159
Low disease activity	11 (13.2)^a^	29 (26.6)^a^	71 (19.3)^a^	6 (13.3)^a^			
Moderate disease activity	34 (41.0)^a^	43 (39.4)^a^	165 (45.0)^a^	19 (42.2)^a^			
High disease activity	5 (6.0)^a^	11 (10.1)^a^	61 (16.6)^a^	17 (37.8)^b^			
Physical activity (IPAQ-SF)							
Low	42 (50.6)^a^	56 (51.4)^a^	228 (62.1)^a, b^	35 (77.8)^b^	18.933	0.004	0.125
Moderate	37 (44.6)^a^	49 (45.0)^a^	135 (36.8)^a, b^	8 (17.8)^b^			
High	4 (4.8)^a^	4 (3.6)^a^	4 (1.1)^a^	2 (4.4)^a^			
Pain intensity (BPI-SF)	2.67 ± 2.79	3.18 ± 2.82	3.41 ± 2.85	4.93 ± 2.82	6.398	< 0.001	0.031
Pain interference (BPI-SF)	9.61 ± 12.97	12.79 ± 14.46	15.57 ± 15.40	29.31 ± 16.78	18.054	< 0.001	0.083
Pain duration (days)	75.16 ± 96.17	51.78 ± 55.43	60.97 ± 76.97	81.29 ± 69.83	2.449	0.063	0.012
Physical health (WHOQOL-BREF)	60.35 ± 15.04	55.95 ± 18.02	53.16 ± 15.95	53.20 ± 20.18	4.603	0.003	0.022
Psychological health (WHOQOL-BREF)	59.21 ± 14.74	56.43 ± 19.31	53.91 ± 17.85	50.42 ± 22.72	3.096	0.026	0.015
Social relationships (WHOQOL-BREF)	60.12 ± 16.11	56.35 ± 18.24	52.71 ± 19.50	48.76 ± 23.36	5.036	0.002	0.025
Environment (WHOQOL-BREF)	58.94 ± 12.04	55.90 ± 14.93	53.91 ± 15.46	53.07 ± 17.72	2.876	0.036	0.014
Social support (SSRS)	38.52 ± 6.12	36.34 ± 5.88	34.75 ± 4.92	32.93 ± 5.83	15.498	< 0.001	0.072
Pain anxiety (PASS)	22.86 ± 19.81	34.51 ± 21.18	44.51 ± 20.35	57.27 ± 18.82	38.955	< 0.001	0.163
Patient activation (PAM-13)	58.49 ± 12.96	64.12 ± 15.15	64.82 ± 15.02	60.11 ± 13.80	5.069	0.002	0.025

Class 1, low cognition-attitude with moderate behavior group; Class 2, low cognition with high attitude-behavior group; Class 3, high cognition with high attitude-behavior group; Class 4, extremely high cognition-attitude-behavior group.

Values are presented as mean ± SD or *n* (%). Percentages were calculated within each latent profile. For categorical variables, superscript letters indicate Bonferroni-adjusted pairwise comparisons of column proportions within the same row; values with different superscript letters differ significantly at *p* < 0.05, whereas values sharing the same letter are not significantly different.Effect size refers to η^2^ for continuous variables and Cramer's V for categorical variables.

BPI-SF, Brief Pain Inventory-short form; WHOQOL-BREF, world health organization quality of life-brief version; SSRS, Social Support Rating Scale; PASS, Pain Anxiety Symptoms Scale; PAM-13, patient activation measure; IPAQ-SF, International Physical Activity Questionnaire-short form; BMI, Body Mass Index; DAS28, disease activity score in 28 joints.

In comparison, Class 1 included a higher proportion of patients in disease remission and those with higher monthly income, and demonstrated generally better quality of life and greater social support. Patient activation also differed significantly across the profiles, with participants in Classes 2 and 3 showing significantly higher activation scores than those in Class 1, as detailed in Table 4.

**Table 4:** *Post hoc* pairwise comparisons of continuous variables across the four latent profiles.

Variable	Pairwise comparison	MD (SE)	*p*	95% *CI*	
				Lower	Upper
Pain intensity (BPI-SF)					
	Class 1 vs. Class 2	−0.51 (0.41)	0.607	−1.57	0.56
	Class 1 vs. Class 3	−0.73 (0.34)	0.147	−1.62	0.16
	Class 1 vs. Class 4	−2.26 (0.52)	< 0.001	−3.61	−0.91
	Class 2 vs. Class 3	−0.22 (0.31)	0.889	−1.02	0.57
	Class 2 vs. Class 4	−1.75 (0.50)	0.003	−3.04	−0.46
	Class 3 vs. Class 4	−1.53 (0.45)	0.004	−2.68	−0.37
Pain interference (BPI-SF)					
	Class 1 vs. Class 2	−3.17 (2.19)	0.469	−8.81	2.47
	Class 1 vs. Class 3	−5.96 (1.83)	0.006	−10.67	−1.25
	Class 1 vs. Class 4	−19.70 (2.78)	< 0.001	−26.86	−12.53
	Class 2 vs. Class 3	−2.79 (1.64)	0.325	−7.01	1.44
	Class 2 vs. Class 4	−16.52 (2.66)	< 0.001	−23.38	−9.66
	Class 3 vs. Class 4	−13.74 (2.37)	< 0.001	−19.85	−7.62
Physical health (WHOQOL-BREF)					
	Class 1 vs. Class 2	4.41 (2.41)	0.262	−1.81	10.62
	Class 1 vs. Class 3	7.19 (2.01)	0.002	2.00	12.38
	Class 1 vs. Class 4	7.15 (3.07)	0.092	−0.75	15.05
	Class 2 vs. Class 3	2.78 (1.81)	0.414	−1.87	7.44
	Class 2 vs. Class 4	2.75 (2.94)	0.785	−4.81	10.31
	Class 3 vs. Class 4	−0.04 (2.62)	1.000	−6.78	6.70
Psychological health (WHOQOL-BREF)					
	Class 1 vs. Class 2	2.78 (2.64)	0.720	−4.03	9.58
	Class 1 vs. Class 3	5.30 (2.20)	0.078	−0.38	10.97
	Class 1 vs. Class 4	8.79 (3.36)	0.045	0.14	17.44
	Class 2 vs. Class 3	2.52 (1.98)	0.580	−2.58	7.62
	Class 2 vs. Class 4	6.01 (3.21)	0.242	−2.27	14.29
	Class 3 vs. Class 4	3.49 (2.86)	0.615	−3.89	10.87
Social relationships (WHOQOL-BREF)					
	Class 1 vs. Class 2	3.77 (2.79)	0.532	−3.43	10.96
	Class 1 vs. Class 3	7.41 (2.33)	0.008	1.41	13.41
	Class 1 vs. Class 4	11.36 (3.55)	0.008	2.22	20.50
	Class 2 vs. Class 3	3.64 (2.09)	0.304	−1.75	9.03
	Class 2 vs. Class 4	7.59 (3.40)	0.115	−1.16	16.34
	Class 3 vs. Class 4	3.95 (3.03)	0.559	−3.85	11.75
Environment (WHOQOL-BREF)					
	Class 1 vs. Class 2	3.04 (2.20)	0.512	−2.64	8.72
	Class 1 vs. Class 3	5.03 (1.84)	0.032	0.29	9.77
	Class 1 vs. Class 4	5.87 (2.80)	0.155	−1.34	13.09
	Class 2 vs. Class 3	1.99 (1.65)	0.624	−2.26	6.24
	Class 2 vs. Class 4	2.83 (2.68)	0.716	−4.07	9.74
	Class 3 vs. Class 4	0.84 (2.39)	0.985	−5.31	7.00
Social support (SSRS)					
	Class 1 vs. Class 2	2.18 (0.78)	0.027	0.17	4.19
	Class 1 vs. Class 3	3.76 (0.65)	< 0.001	2.09	5.44
	Class 1 vs. Class 4	5.58 (0.99)	< 0.001	3.03	8.14
	Class 2 vs. Class 3	1.58 (0.58)	0.034	0.08	3.09
	Class 2 vs. Class 4	3.41 (0.95)	0.002	0.97	5.85
	Class 3 vs. Class 4	1.82 (0.84)	0.137	−0.35	4.00
Pain anxiety (PASS)					
	Class 1 vs. Class 2	−11.66 (2.96)	< 0.001	−19.29	−4.03
	Class 1 vs. Class 3	−21.65 (2.47)	< 0.001	−28.02	−15.29
	Class 1 vs. Class 4	−34.41 (3.76)	< 0.001	−44.10	−24.72
	Class 2 vs. Class 3	−9.99 (2.22)	< 0.001	−15.70	−4.28
	Class 2 vs. Class 4	−22.75 (3.60)	< 0.001	−32.03	−13.48
	Class 3 vs. Class 4	−12.76 (3.21)	< 0.001	−21.03	−4.49
Patient activation (PAM-13)					
	Class 1 vs. Class 2	−5.63 (2.14)	0.043	−11.14	−0.11
	Class 1 vs. Class 3	−6.33 (1.79)	0.002	−10.94	−1.73
	Class 1 vs. Class 4	−1.63 (2.72)	0.933	−8.63	5.38
	Class 2 vs. Class 3	−0.71 (1.60)	0.971	−4.84	3.42
	Class 2 vs. Class 4	4.00 (2.60)	0.416	−2.70	10.71
	Class 3 vs. Class 4	4.71 (2.32)	0.179	−1.27	10.69

MD, mean difference; SE, standard error; BPI-SF, Brief Pain Inventory-short form; WHOQOL-BREF, world health organization quality of life-brief version; SSRS, Social Support Rating Scale; PASS, Pain Anxiety Symptoms Scale; PAM-13, patient activation measure; BMI, Body Mass Index; DAS28, Disease Activity Score in 28 joints. Class 1, low cognition-attitude with moderate behavior group; Class 2, low cognition with high attitude-behavior group; Class 3, high cognition with high attitude-behavior group; Class 4, extremely high cognition-attitude-behavior group.

## 4 Discussion

This study identified four latent profiles of kinesiophobia among patients with RA, suggesting that kinesiophobia is not merely distributed along a single severity continuum but reflects heterogeneous patterns of activity-related cognition, attitude, and behavior. Class 1 was characterized by low cognitive and attitudinal scores with moderate behavioral scores. Class 2 showed relatively low cognitive scores but high attitudinal and behavioral scores, indicating a discordant cognition–attitude/behavior profile. In contrast, Class 3, the largest subgroup, showed high scores in cognition, attitude, and behavior, whereas Class 4 had the highest scores across all three dimensions. The main distinction between Class 2 and Class 3 was the cognitive dimension: although both classes showed high attitudinal and behavioral scores, Class 3 additionally demonstrated elevated cognitive scores. Therefore, the separation between Class 2 and Class 3 was supported not only by statistical model selection but also by differences in cognitive threat appraisal related to movement. Given the cross-sectional design, these latent profiles should be interpreted as coexisting patterns at a single time point rather than as evidence of causal relationships, temporal ordering, or stage-like progression. The stability of these profiles and their potential changes over time should be examined in future longitudinal studies.

Gender, monthly income, and social support differed significantly across the identified profiles, suggesting that sociodemographic characteristics and social resources may influence the development of distinct patterns of kinesiophobia in patients with RA. The higher proportion of women in Class 4 should be interpreted with caution because the observed effect size was small and the study population was pre-dominantly female. Patients with lower monthly income were more frequently represented in the higher-risk profiles, particularly Class 4, whereas those with higher income were more commonly classified into Class 1. These results are consistent with previous studies indicating that individuals with lower socioeconomic status often have reduced access to disease management resources, rehabilitation services, and health-related information (29, 30).

Social support was highest in Class 1 and lowest in Class 4, consistent with evidence showing that strong social support enhances coping ability and encourages positive health behaviors (31–33). Emotional, informational, and practical support may alleviate excessive perceptions of activity-related risk and promote confidence in engaging safely in physical activity (31). These results suggest that the management of kinesiophobia in patients with RA should extend beyond individual-focused health education to include greater involvement of family members, caregivers, and broader social support networks. In addition, improving access to healthcare resources and supportive services for patients with lower socioeconomic status may help reduce disparities in kinesiophobia and facilitate better disease self-management.

Disease activity, pain intensity, pain interference, and pain anxiety collectively represent key clinical and psychological factors associated with kinesiophobia in patients with RA. Patients in Class 4 showed higher disease activity, a greater pain burden, and more severe pain anxiety, whereas those in Class 1 demonstrated a comparatively more favorable clinical profile. These results are in good agreement with previous studies reporting significant associations between kinesiophobia and disease activity, pain severity, and pain interference in patients with RA (31, 34). Increased disease activity may amplify perceived movement-related risk by exacerbating pain, joint swelling, fatigue, and post-activity discomfort (4). Similarly, pain interference may repeatedly reinforce the association between physical activity and pain or functional difficulty, therefore strengthening avoidance behaviors (4, 31).

Pain anxiety demonstrated the most pronounced gradient across the four profiles, with all pairwise comparisons reaching statistical significance. This aligns with the fear-avoidance model and suggests that emotional responses to pain and perceived movement-related risk play a pivotal role in differentiating patterns of kinesiophobia (34, 35). These results highlight the importance of a comprehensive nursing assessment that extends beyond evaluating pain intensity alone to include patients' concerns, strong vigilance, and catastrophic beliefs regarding pain exacerbation, joint damage, and the perceived risks associated with movement.

Physical activity and quality of life also varied significantly across the identified profiles. Patients in Class 4 reported lower levels of physical activity and poorer quality of life, whereas those in Class 1 demonstrated relatively better quality of life. *Post hoc* comparisons showed that Class 1 had significantly higher physical health and environment scores than Class 3, higher psychological health scores than Class 4, and higher social relationship scores than Classes 3 and 4. These results are consistent with previous studies in patients with RA showing that kinesiophobia is associated with insufficient physical activity and diminished quality of life, suggesting that fear of movement is not merely a psychological response but is closely linked to actual activity participation and everyday living experiences (12, 31).

Patient activation also differed significantly across the identified profiles, with participants in Classes 2 and 3 demonstrating significantly higher activation scores than those in Class 1. Previous studies on self-management in RA have shown that greater patient activation is generally associated with better disease management and healthier behaviors (20, 29). However, in the present study, higher patient activation was not consistently associated with lower kinesiophobia. This finding should be interpreted cautiously. Given the cross-sectional design, the direction of this association cannot be determined. It is possible that patients with greater symptom burden, higher disease activity, or more frequent disease-management needs may report higher activation, while these same clinical factors may also be associated with greater fear of movement. Therefore, reverse causality and confounding by disease severity or pain-related factors cannot be excluded. From a clinical perspective, these findings suggest that patient activation should be interpreted in relation to patients' clinical status, pain burden, risk perceptions, and actual physical activity behavior. Future longitudinal studies are needed to clarify how patient activation is related to changes in kinesiophobia and movement behavior over time.

From a nursing practice perspective, these results support a stratified approach to the assessment and management of kinesiophobia in patients with RA. Nurses can use brief screening instruments such as the TSK-11 and combine the results with information on sociodemographic characteristics, social support, disease activity, pain burden, pain anxiety, physical activity, quality of life, and patient activation to conduct a comprehensive assessment.

For patients in Class 1, nursing care should emphasize maintaining safe participation in physical activity while preventing unnecessary activity restriction. For those in Class 2, early interventions should focus on addressing negative attitudes toward movement and reducing avoidance behaviors (29, 36). In Class 3, nursing strategies should prioritize restructuring movement-related threat perceptions while guiding patients with higher levels of activation toward evidence-based exercise and self-management practices (37, 38). Patients in Class 4 are likely to benefit from more intensive multidisciplinary care that integrates pain management, psychological support, exercise rehabilitation, physical activity promotion, and strategies to strengthen social support (39, 40).

Tailoring nursing interventions to the distinct cognitive, attitudinal, and behavioral characteristics of each kinesiophobia profile may improve the precision of clinical management, promote safe engagement in physical activity, and ultimately enhance the quality of life of patients with RA.

Several limitations of this study should be acknowledged. First, the cross-sectional design precludes causal inference; longitudinal studies are needed to investigate changes and potential transitions in kinesiophobia profiles over time. Second, participants were recruited using convenience sampling from three tertiary Grade A hospitals in Henan Province, China. Therefore, the sample may overrepresent patients receiving tertiary rheumatology care in a single geographic and healthcare context. This limits the generalizability of the findings to patients in primary care or community settings, other regions of China, and other countries or healthcare systems. Furthermore, most study variables were assessed using self-reported measures, which are subject to reporting bias. Future studies should incorporate longitudinal designs, objective assessments of physical activity and behavior, and external validation cohorts to strengthen the validity and generalizability of these findings.

## 5 Conclusion

This study identified four distinct latent profiles of kinesiophobia among patients with RA, highlighting the heterogeneous cognitive, attitudinal, and behavioral patterns underlying fear of movement. These profiles differed in their sociodemographic, clinical, psychosocial, and self-management characteristics, with pain anxiety demonstrating the most pronounced gradient across profiles and patient activation showing a more complex relationship with kinesiophobia. These results support the use of profile-based, stratified nursing interventions to provide more individualized management, reduce fear of movement, and promote safe participation in physical activity among patients with RA.

## Data Availability

The original contributions presented in the study are included in the article/supplementary material, further inquiries can be directed to the corresponding authors.

## References

[1] Maisha JA El-Gabalawy HS O'Neil LJ. Modifiable risk factors linked to the development of rheumatoid arthritis: evidence, immunological mechanisms and prevention. Front Immunol. (2023) 14:1221125. doi: 10.3389/fimmu.2023.1221125 37767100 PMC10520718

[2] Bilberg A Mannerkorpi K Borjesson M Svedlund S Sivertsson J Klingberg E . High-intensity interval training improves cardiovascular and physical health in patients with rheumatoid arthritis: a multicentre randomised controlled trial. Br J Sports Med. (2024) 58:1409–18. doi: 10.1136/bjsports-2024-108369 39179363 PMC11672065

[3] Steultjens M Bell K Hendry G. The challenges of measuring physical activity and sedentary behaviour in people with rheumatoid arthritis. Rheumatol Adv Pract. (2023) 7:rkac101. doi: 10.1093/rap/rkac101 36699550 PMC9870705

[4] Reinoso-Cobo A Ortega-Avila AB Ramos-Petersen L García-Campos J Banwell G Gijon-Nogueron G . Relationship between kinesiophobia, foot pain and foot function, and disease activity in patients with rheumatoid arthritis: a cross-sectional study. Medicina (Kaunas). (2023) 59:147. doi: 10.3390/medicina59010147 36676771 PMC9864291

[5] Vlaeyen JWS Crombez G Linton SJ. The fear-avoidance model of pain. Pain. (2016) 157:1588–9. doi: 10.1097/j.pain.0000000000000574 27428892

[6] Xueying S Yanli Y Wei X Lingling Z Lili L. Influence of kinesiophobia on activity, function, and anxiety levels in patients with rheumatoid arthritis. Front Med (Lausanne). (2024) 11:1514088. doi: 10.3389/fmed.2024.1514088 39882524 PMC11774740

[7] Băjenaru L Balog A Dobre C Drăghici R Prada G-I. Latent profile analysis for quality of life in older patients. BMC Geriatr. (2022) 22:848. doi: 10.1186/s12877-022-03518-1 36368920 PMC9652949

[8] Ying L Yuyu D Daili Z Yangmei S Qing X Zhihuan Z. Latent profile analysis of missed nursing care and their predictors among neuro-oncology nurses: a multicenter cross-sectional study. BMC Nurs. (2025) 24:419. doi: 10.1186/s12912-025-03094-w 40229749 PMC11998220

[9] van der Linden MPM Knevel R Huizinga TWJ van der Helm-van Mil AHM. Classification of rheumatoid arthritis: comparison of the 1987 American college of rheumatology criteria and the 2010 American college of rheumatology/european league against rheumatism criteria. Arthritis Rheum. (2011) 63:37–42. doi: 10.1002/art.30100 20967854

[10] Alpalhão V Vaz JR Cordeiro N de Pezarat Correia P. Are the Cross-Culturally Adapted versions of the tampa scale for kinesiophobia 11-item valid, reliable, and responsive? A COSMIN-informed systematic review of measurement properties. J Pain. (2024) 25:104602. doi: 10.1016/j.jpain.2024.104602 38866123

[11] Woby SR Roach NK Urmston M Watson PJ. Psychometric properties of the TSK-11: a shortened version of the tampa scale for kinesiophobia. Pain. (2005) 117:137–44. doi: 10.1016/j.pain.2005.05.029 16055269

[12] Liu H Li H Huang L Tian H Wu J Guan Q . The link between childhood physical activity enjoyment and adult kinesiophobia in individuals with chronic low back pain. BMC Public Health. (2024) 24:2557. doi: 10.1186/s12889-024-19953-1 39300388 PMC11414032

[13] Cai L Liu Y Woby SR Genoosha N Cui M Guo L. Cross-cultural adaptation, reliability, and validity of the chinese version of the tampa scale for kinesiophobia-11 among patients who have undergone total knee arthroplasty. J Arthroplasty. (2019) 34:1116–21. doi: 10.1016/j.arth.2019.01.076 30853160

[14] Fizazi K Herrmann K Krause BJ Rahbar K Chi KN Morris MJ . Health-related quality of life and pain outcomes with [177Lu]Lu-PSMA-617 plus standard of care versus standard of care in patients with metastatic castration-resistant prostate cancer (VISION): a multicentre, open-label, randomised, phase 3 trial. Lancet Oncol. (2023) 24:597–610. doi: 10.1016/S1470-2045(23)00158-4 37269841 PMC10641914

[15] Chiarotto A Maxwell LJ Ostelo RW Boers M Tugwell P Terwee CB. Measurement properties of visual analogue scale, numeric rating scale, and pain severity subscale of the brief pain inventory in patients with low back pain: a systematic review. J Pain. (2019) 20:245–63. doi: 10.1016/j.jpain.2018.07.009 30099210

[16] McCracken LM Dhingra L. A short version of the pain anxiety symptoms scale (PASS-20): preliminary development and validity. Pain Res Manag. (2002) 7:45–50. doi: 10.1155/2002/517163 16231066

[17] Vowles KE Kruger ES Bailey RW Ashworth J Hickman J Sowden G . The pain anxiety symptom scale: initial development and evaluation of 4 and 8 item short forms. J Pain. (2024) 25:176–86. doi: 10.1016/j.jpain.2023.08.001 37574179

[18] Zhou X-Y Xu X-M Wang F Wu S-Y Yang Y-L Li M . Validations and psychological properties of a simplified Chinese version of pain anxiety symptoms scale (SC-PASS). Medicine (Baltimore). (2017) 96:e5626. doi: 10.1097/MD.0000000000005626 28272194 PMC5348142

[19] Wenhong A Xuefeng T Xueling X Waha A Honghong W. Status and factors associated with patient activation and its relationship with HIV clinic outcomes among Yi minority people living with HIV in Liangshan, China: a cross-sectional study. Front Public Health. (2023) 11:1114561. doi: 10.3389/fpubh.2023.1114561 37397752 PMC10309002

[20] Sui W Wan L-H. Association between patient activation and medication adherence in patients with stroke: a cross-sectional study. Front Neurol. (2021) 12:722711. doi: 10.3389/fneur.2021.722711 34659088 PMC8516066

[21] Wang J Wang Q Bao Z Peng Y Liu S Yu T . The combined effects of patient activation and relational aspects on the quality of life in atrial fibrillation patients. Front Psychol. (2021) 12:761149. doi: 10.3389/fpsyg.2021.761149 34867661 PMC8636326

[22] Zhan T Li X Wang Y Wang L Sun C Hu X . Social support, self-management behaviors, and coping styles of patients with Wilson's disease: a quantitative empirical research. Brain Behav. (2025) 15:e71138. doi: 10.1002/brb3.71138 41431312 PMC12723061

[23] Wang X Zhang F Ge Y Ding Y Liu T. The associations between social support, self-regulatory fatigue, and health-promoting behaviors among people with type 2 diabetes mellitus: a cross-sectional survey. Front Public Health. (2023) 11:1281065. doi: 10.3389/fpubh.2023.1281065 38155890 PMC10752976

[24] Wang X Wu Y Meng Z Li J Xu L Sun X . Willingness to use mobile health devices in the Post-COVID-19 era: nationwide cross-sectional study in China. J Med Internet Res. (2023) 25:e44225. doi: 10.2196/44225 36719823 PMC9942786

[25] Nikolic A Bukurov B Kocic I Vukovic M Ladjevic N Vrhovac M . Smartphone addiction, sleep quality, depression, anxiety, and stress among medical students. Front Public Health. (2023) 11:1252371. doi: 10.3389/fpubh.2023.1252371 37744504 PMC10512032

[26] Flora S Marques A Hipólito N Morais N Silva CG Januário F . Test-retest reliability, agreement and construct validity of the International Physical Activity Questionnaire short-form (IPAQ-sf) in people with COPD. Respir Med. (2023) 206:107087. doi: 10.1016/j.rmed.2022.107087 36525854

[27] Sheng Y Shen F Xiong M Huang Q Li Q Hu L. Physical activity levels and influencing factors among colorectal cancer patients: a cross-sectional study. BMC Psychol. (2025) 13:171. doi: 10.1186/s40359-025-02541-2 40022243 PMC11869429

[28] Gil-Lacruz M Cañete-Lairla M Navarro J Montaño-Espinoza R Espinoza-Santander I Osorio-Parraguez P. Validation of the WHOQOL-BREF quality of life questionnaire in an urban sample of older adults in a neighbourhood in Zaragoza (Spain). Healthcare (Basel). (2022) 10:2272. doi: 10.3390/healthcare10112272 36421596 PMC9690437

[29] Bordeleau M Vincenot M Lefevre S Duport A Seggio L Breton T . Treatments for kinesiophobia in people with chronic pain: a scoping review. Front Behav Neurosci. (2022) 16:933483. doi: 10.3389/fnbeh.2022.933483 36204486 PMC9531655

[30] Perez-Dominguez B Perpiña-Martinez S Garcia-Isidoro S Escobio-Prieto I Rodriguez-Rodriguez AM Blanco-Diaz M. Associations between preoperative patient socioeconomic status and pain-related outcomes with pain and function in patients undergoing rotator cuff repairs. Healthcare (Basel). (2023) 11:2786. doi: 10.3390/healthcare11202786 37893860 PMC10606215

[31] Liu L Yang Q Li T Xie H Zeng B Zha L . Prevalence and influencing factors of kinesiophobia in patients with heart disease: a meta-analysis and systematic review. Sci Rep. (2024) 14:18956. doi: 10.1038/s41598-024-69929-9 39147837 PMC11327283

[32] Du X Shao Y Xue J Kong J. Prevalence and influencing factors of kinesiophobia after total knee arthroplasty: a systematic review and meta-analysis. J Orthop Surg Res. (2025) 20:332. doi: 10.1186/s13018-025-05752-w 40170179 PMC11959722

[33] Marmouta P Marmouta L Tsounis A Tzavara C Malliarou M Fradelos E . Effect of kinesiophobia and social support on quality of life after total hip arthoplasty. Healthcare (Basel). (2025) 13:1366. doi: 10.3390/healthcare13121366 40565392 PMC12193136

[34] Wood L Bejarano G Csiernik B Miyamoto GC Mansell G Hayden JA . Pain catastrophising and kinesiophobia mediate pain and physical function improvements with Pilates exercise in chronic low back pain: a mediation analysis of a randomised controlled trial. J Physiother. (2023) 69:168–74. doi: 10.1016/j.jphys.2023.05.008 37277290

[35] Vlaeyen JWS Kole-Snijders AMJ Boeren RGB van Eek H. Fear of movement/(re)injury in chronic low back pain and its relation to behavioral performance. Pain. (1995) 62:363–72. doi: 10.1016/0304-3959(94)00279-N 8657437

[36] Doutre L Beaumier M Parent A-A Talbot S Tremblay M. Kinesiophobia among health professionals' interventions: a scoping review. PeerJ. (2024) 12:e17935. doi: 10.7717/peerj.17935 39184383 PMC11344999

[37] Cai S Yao J Han M Luo X Yu Y Lu X . The effect of cognition in combination with an ACBT on dyspnea-related kinesiophobia in patients with moderate to severe COPD: Quasirandomized controlled trial study. Geriatr Nurs. (2024) 56:138–47. doi: 10.1016/j.gerinurse.2024.01.002 38342002

[38] Zhu S Xu Y Wang L Chen J Luo A. Efficacy of cognitive behavioral therapy for kinesiophobia: a systematic review and meta-analysis of randomized controlled trials. J Pain Res. (2025) 18:3919–32. doi: 10.2147/JPR.S526179 40791870 PMC12336382

[39] Tzu-Han H Mei-Chun L Ya-Wen K Jiann-Der L. Comparative analysis of multidisciplinary complementary intervention versus routine physiotherapy rehabilitatito reduce kinesiophobia in patients with knee osteoarthritis undergoing total knee replacement: a systematic review and meta-analysis. Sports Med Open. (2025) 11:91. doi: 10.1186/s40798-025-00899-6 40770481 PMC12328877

[40] Li C Lin Y Xiao X Guo X Fei J Lu Y . Development and validation of a risk prediction model for kinesiophobia in postoperative lung cancer patients: an interpretable machine learning algorithm study. Sci Rep. (2025) 15:19412. doi: 10.1038/s41598-025-03575-7 40461518 PMC12134359

