# Supplementary material for: Latent profiles of kinesiophobia and associated factors in patients with rheumatoid arthritis: a cross-sectional study
Source: Front Med (Lausanne). 2026 Jul 31;13:1868172. doi: 10.3389/fmed.2026.1868172 (PMC13472796; doi:10.3389/fmed.2026.1868172)
Supplement: Supplementary file 1 [file Data_Sheet_1.docx]

Supplementary Material

Table S1 Mean item scores of the TSK-11 across the four latent profiles

| Item | Class1  (n=83, Mean ± SE) | Class2  (n=109, Mean ± SE) | Class3  (n=367, Mean ± SE) | Class4  (n=45, Mean ± SE) |
| --- | --- | --- | --- | --- |
| Q1 | 1.12 ± 0.04 | 2.80 ± 0.06 | 2.72 ± 0.04 | 3.73 ± 0.08 |
| Q2 | 1.01 ± 0.01 | 1.72 ± 0.05 | 2.66 ± 0.03 | 3.29 ± 0.09 |
| Q3 | 1.00 ± 0.00 | 1.66 ± 0.05 | 2.64 ± 0.03 | 3.49 ± 0.09 |
| Q4 | 1.02 ± 0.02 | 1.50 ± 0.05 | 2.36 ± 0.04 | 3.58 ± 0.09 |
| Q5 | 1.01 ± 0.01 | 1.48 ± 0.05 | 2.47 ± 0.04 | 2.87 ± 0.14 |
| Q6 | 1.01 ± 0.01 | 1.61 ± 0.06 | 3.00 ± 0.02 | 3.82 ± 0.06 |
| Q7 | 2.76 ± 0.11 | 3.15 ± 0.04 | 2.82 ± 0.03 | 3.89 ± 0.05 |
| Q8 | 1.04 ± 0.02 | 1.74 ± 0.06 | 2.87 ± 0.04 | 3.64 ± 0.08 |
| Q9 | 2.70 ± 0.12 | 3.13 ± 0.04 | 2.92 ± 0.03 | 3.78 ± 0.07 |
| Q10 | 2.52 ± 0.12 | 2.95 ± 0.06 | 3.05 ± 0.03 | 3.84 ± 0.06 |
| Q11 | 1.13 ± 0.04 | 2.85 ± 0.04 | 2.92 ± 0.03 | 3.69 ± 0.07 |

Note: Class 1, Low Cognition–Attitude with Moderate Behavior Group; Class 2, Low Cognition with High Attitude–Behavior Group; Class 3, High Cognition with High Attitude–Behavior Group; Class 4, Extremely High Cognition–Attitude–Behavior Group. SE, standard error.

Table S2. Dimension Mean Scores of the TSK-11 Across the Four Latent Profiles

| Dimension | Class1  (n=83, Mean ± SE) | Class2  (n=109, Mean ± SE) | Class3  (n=367, Mean ± SE) | Class4  (n=45, Mean ± SE) |
| --- | --- | --- | --- | --- |
| Activity cognition | 1.02 ± 0.01 | 1.62 ± 0.03 | 2.67 ± 0.01 | 3.45 ± 0.04 |
| Activity attitude | 1.13 ± 0.03 | 2.83 ± 0.04 | 2.82 ± 0.02 | 3.71 ± 0.05 |
| Activity behavior | 2.66 ± 0.10 | 3.08 ± 0.03 | 2.93 ± 0.02 | 3.84 ± 0.04 |

Note: Class 1, Low Cognition–Attitude with Moderate Behavior Group; Class 2, Low Cognition with High Attitude–Behavior Group; Class 3, High Cognition with High Attitude–Behavior Group; Class 4, Extremely High Cognition–Attitude–Behavior Group. Dimension scores were calculated as the mean of the corresponding item scores: Cognitive dimension score = (Q2 + Q3 + Q4 + Q5 + Q6 + Q8) / 6; Attitudinal dimension score = (Q1 + Q11) / 2; Behavioral dimension score = (Q7 + Q9 + Q10) / 3. SE, standard error.
